# A Non-Canonical NRPS Is Involved in the Synthesis of Fungisporin and Related Hydrophobic Cyclic Tetrapeptides in *Penicillium chrysogenum*

**DOI:** 10.1371/journal.pone.0098212

**Published:** 2014-06-02

**Authors:** Hazrat Ali, Marco I. Ries, Peter P. Lankhorst, Rob A. M. van der Hoeven, Olaf L. Schouten, Marek Noga, Thomas Hankemeier, Noël N. M. E. van Peij, Roel A. L. Bovenberg, Rob J. Vreeken, Arnold J. M. Driessen

**Affiliations:** 1 Molecular Microbiology, Groningen Biomolecular Sciences and Biotechnology Institute, Zernike Institute for Advanced Materials, University of Groningen, Groningen, The Netherlands; 2 Kluyver Centre for Genomics of Industrial Fermentations, Delft, The Netherlands; 3 Division of Analytical Biosciences, Leiden Academic Centre for Drug Research, Leiden University, Leiden, The Netherlands; 4 DSM Biotechnology Center, Delft, The Netherlands; 5 Netherlands Metabolomics Centre, Leiden University, Leiden, The Netherlands; 6 Synthetic Biology and Cell Engineering, Groningen Biomolecular Sciences and Biotechnology Institute, University of Groningen, Groningen, The Netherlands; Technical University of Denmark, Denmark

## Abstract

The filamentous fungus Penicillium chrysogenum harbors an astonishing variety of nonribosomal peptide synthetase genes, which encode proteins known to produce complex bioactive metabolites from simple building blocks. Here we report a novel non-canonical tetra-modular nonribosomal peptide synthetase (NRPS) with microheterogenicity of all involved adenylation domains towards their respective substrates. By deleting the putative gene in combination with comparative metabolite profiling various unique cyclic and derived linear tetrapeptides were identified which were associated with this NRPS, including fungisporin. In combination with substrate predictions for each module, we propose a mechanism for a ‘trans-acting’ adenylation domain.

## Introduction

Fungal non-ribosomal peptides contribute a large variety of secondary metabolites with remarkable properties such as antibacterial, antifungal, antiparasitic, anticancer and immunosuppressive activities. These metabolites are produced by large, multifunctional protein complexes, called nonribosomal peptide synthetases (NRPS). These enzymes catalyze the stepwise condensation of simple amino acid building blocks to complex molecules. NRPSs have a modular organization, with each module responsible for one discrete chain-elongation step. Every single module can be subdivided into domains that carry all essential information for recognition, activation and modification of the corresponding substrate. At a minimum, a typical NRPS module consists of an adenylation (A) domain, responsible for amino acid activation, a thiolation domain, also known as peptidyl carrier protein (PCP), which binds the activated amino acid and a condensation (C) domain that catalyzes peptide-bond formation. The common arrangements of these domains follow a (C-A-PCP)_n_ organization. Additionally, a variety of optional domains have been described such as methyltransferase (MT) and epimerization (E) domains [1].

The number of modules and their domain organization within NRPS enzymes controls the structures of the final product(s) [1]–[3]. Thus, the order of modules usually corresponds to the sequence of amino acids in the peptide. Many NRPS systems adhere to this mechanistic paradigm, which is often referred to as the “co-linearity rule” [4]. Also exceptions to this rule have been discovered, including iterative NRPSs, which incorporate multiple residues of the same amino acid iteratively into the peptide structure and the so called nonlinear NRPSs, which deviate completely from the standard domain organization leading to unexpected products [3], [5], [6].

The impact of non-ribosomal peptide metabolites on the quality of human life raised the interest of pharmaceutical industries to invest in identification, engineering and heterologous expression of NRPS genes and pathways to ensure the rational production of novel compounds [7]–[9]. To understand the basic mechanisms of the biosynthesis of these complex NRPSs, detailed studies have been performed during the past few decades. These included the structural analysis of adenylation domains, mutational analysis of substrate specificity of these modules, the fusion of unrelated modules to produce new products and the identification of helper proteins for optimal activation of adenylation domains [10]–[12]. Although this has led to detailed insights into catalytic mechanisms, so far a structure of a complete NRPS is lacking that would reveal how modules cooperate to facilitate product formation. The availability of genome sequencing data and sophisticated bioinformatics analysis of various fungi revealed the presence of many NRPS genes that have not been associated with known secondary metabolites [13]–[15]. Moreover, most of these genes are not expressed when the fungi are grown under laboratory conditions, implying that many more secondary metabolites await discovery.

**Figure 1:**
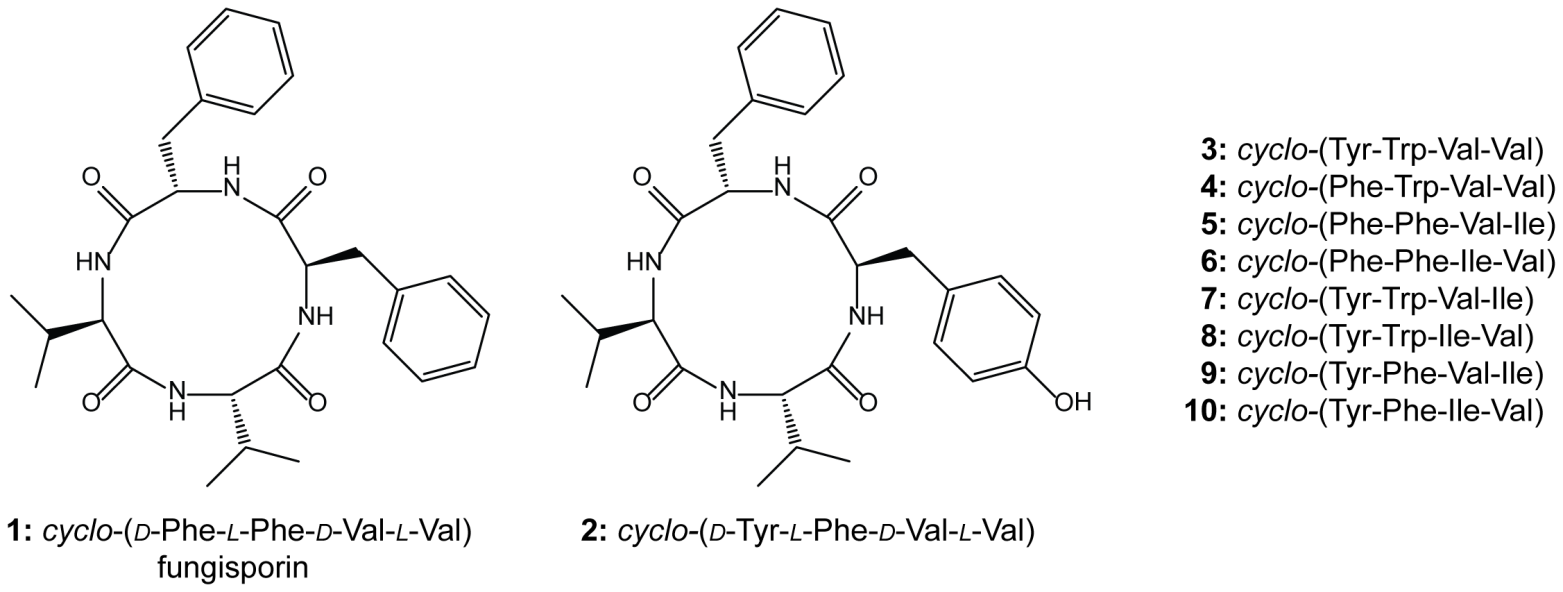
Identified secondary metabolites. Structures of cyclic tetrapeptides identified in *P. chrysogenum*.

The filamentous fungus *Penicillium chrysogenum* is well known for the production of the antibiotic penicillin G that is synthesized by the tri-modular NRPS δ-(L-α-aminoadipyl)-L-cysteinyl-D-valine synthetase. In addition, other NRPS derived secondary metabolites like the roquefortines and meleagrin have been reported from *P. chrysogenum* [16]–[18]. Here, we describe the identification and structural characterization of cyclic tetrapeptides (Figure 1), including the previously identified metabolite fungisporin [19], [20], and the discovery of a tetra-modular NRPS with an unusual domain organization with adenylation domains showing microheterogenicity. It is proposed to term this NRPS HcpA (CAP93139.1) based on the produced Hydrophobic cyclic peptides.

## Materials and Methods

### A. Chemicals

6-aminoquinolyl-N-hydroxysuccinimidyl carbamate (AQC) reagent and borate buffer were obtained as part of AccQ Tag Reagent Kit from Waters (Waters, Milford, MA, USA). *cyclo-*(*d*-Tyr-*l*-Phe-*d*-Val-*l*-Val) was obtained from Celtek Peptides (Nashville, TN).

### B. Host strains, media, grown condition and plasmid construction

Deletion of the *hcpA* gene was carried out in *P. chrysogenum* strain DS54555, which lacks penicillin cluster genes and the *ku70* gene [18]. This strain was kindly provided by the DSM Biotechnology Center (Delft, Netherlands). A deletion plasmid was constructed by amplifying the flanking regions of the targeted gene with the Multisite Gateway^®^Three-Fragment Vector Construction Kit according to the procedure described by Invitrogen using pDEST R_4_-R_3_p as template. Primers used for the construction of the deletion plasmid pDEST R_4_-R_3_p PcHcpA (Figure S1) are listed in Table S1. *Escherichia coli* DH5α (F– Φ80*lacZ*ΔM15 Δ(*lacZYA-argF*) U169 *recA*1 *endA*1 *hsdR*17 (rK–, mK+) *phoA supE*44 λ– *thi*-1 *gyrA*96 *relA*1) was used as host strain for high frequency transformation and plasmid DNA amplification [21]. All the strains were grown on yeast nitrogen base-glucose-yeast extract (YGG)-medium for protoplasts formation and transformation [22]. Both mutant and host strains of *P. chrysogenum* were grown on secondary metabolite production medium as described previously [18].

### C. Transformation procedure

The deletion plasmid pDes R4-R3p PcHcpA was transformed to the protoplasts of *P. chrysogenum* DS54555 [23] yielding the Δ*hcpA* derivative of strain DS54555 The phleomycin resistance gene was used as selection marker for the deletion of the *HcpA* gene [22], [24].

### D. Genomic DNA extraction, total RNA extraction, cDNA amplification *and qPCR analysis*

Genomic DNA (gDNA) was isolated after 96 hours of growth on SMP medium (secondary metabolite production medium) using the modified yeast gDNA isolation protocol [25] in which the fungal mycelium is broken in a FastPrep FP120 system (Qbiogene). Isolated gDNA was measured using a NanoDrop ND-1000. gDNA of the host and various deletion strains was isolated using the E.Z.N.A. Fungal DNA kit (Omega Bio-tek). Total RNA of the host strain was isolated after 48 hours of growth in SMP medium for the first time and then with the interval of 24 hours up to 216 hours of growth using the Trizol reagent (Invitrogen), with additional DNase treatment using the Turbo DNA-free kit (Ambion). Total RNA was measured with the NanoDrop ND-1000 and a concentration of 500 ng per cDNA reaction was used. cDNA was synthesized using the iScript cDNA synthesis kit (Bio-Rad) in a 10 µl end volume. The primers used to analyze the expression of the hcpA gene were designed around an intron to avoid amplification on gDNA (Table S2). For expression analyses, the γ-actin gene was used as a control for normalization (Table S2). A negative reverse transcriptase (RT) control was used to determine the gDNA contamination in isolated total RNA. The expression levels were determined as described [18].

### E. Southern blotting

Southern blotting was carried out by digesting gDNA (5 µg) with the indicated restriction enzymes. Digested DNA fragments were separated on a 0.8 % agarose gel, blotted onto a Zeta-Probe membrane (Biorad) as described earlier [26], and hybridized with the indicated DIG labeled probes.

### F. Metabolite profiling and analysis

#### 1. Sample preparation

Host and deletion strains of P. chrysogenum strains used for gene assignments were grown in quintuplicate according to the procedure described above. Samples for acquisition of the metabolite profiles from the growth curves were from five replicates. Metabolite profiling was carried out with modifications as described earlier [18]. Briefly, 4 µl of internal standard mixture (855 nmol/mL ranitidine, 657 nmol/mL reserpine and 114 nmol/mL ampicillin) was added to 100 µl fermentation broth followed by the addition of 400 µl methanol for protein precipitation. The samples were vortexed and spun down at 14,000 g for 10 minutes. 300 µL supernatant was evaporated for 30 minutes in a speedvac (Thermo Scientific, San Jose, CA) and re-dissolved in 100 µL water. LC-UV-MS analysis was performed on an Agilent 1200 Capillary pump (Agilent, Santa Clara, CA) coupled in-line to a Surveyor PDA detector (Thermo Scientific, San Jose, CA) and LTQ-FT mass spectrometer (Thermo Scientific, San Jose, CA) using electrospray ionization and operated in a scan range between m/z 110 and m/z 2000 in positive/negative ion switching mode. Separation was performed on a Waters Atlantis T3 column (2.1×100 mm, 3 µm) (Waters, Milford, MA) starting with 98 % of solvent A (1 % acetonitrile and 0.1 % formic acid in water) and 2 % solvent B (1 % water and 0.1 % formic acid in acetonitrile) for 1.5 minutes at a flow rate of 300 µL/min. 40 % B were reached after 22 minutes and 100 % B at 25 minutes. The column was flushed with 100 % B and re-equilibrated to initial conditions. Peak detection and integration were performed using an in-house tool followed by statistical tests to discover significant different features. Finally, discovered features were integrated using LCquan v.26 (Thermo Scientific, San Jose, CA). The non-related non-endogenous compound reserpine was used as internal standard.

#### 2. Identification of cyclic tetrapeptides

The identity of cyclic tetrapeptides was determined using samples from liquid cultures of *P. chrysogenum* and a crude spontaneous precipitate obtained from *P. chrysogenum* cultures, containing primarily **1** and **2** next to various minor abundant cyclic tetrapeptides in relative concentrations of 70 and 15 %, respectively. LC-MS^n^ experiments for the determination of consecutive amino acid losses were performed according the metabolite profiling section with normalized collision energies of 35 %, an isolation width of 1 amu and an activation Q of 0.30. NMR spectra were recorded on a Bruker Avance III 700 MHz NMR spectrometer (Bruker, Billerica, MA), equipped with a 5 mm TCI probe. 2 mg of each sample was dissolved in 0.6 mL anhydrous DMSO. NMR spectra were acquired at 340 K.

#### 3. Identification of linear tetrapeptides

Linear tetrapeptides were identified according their multiple-stage fragmentation after AQC derivatization [27]. Methanol (400 µL) was added to an aliquot of 100 µL fermentation broth for protein precipitation. Samples were vortexed for 10 minutes, spun down for 10 minutes and 300 µL of the supernatant was evaporated to dryness in a speedvac (Thermo Scientific, San Jose, CA). Derivatization was done according to the supplier's procedure by re-dissolving the sample in 40 µL water, 40 µL borate buffer (pH 8.5) and 20 µL AQC solution. The mixture was vortexed for 10 minutes and heated for 10 minutes at 55°C.

LC-MS^n^ experiments were conducted on an Agilent 1200 Capillary pump (Agilent, Santa Clara, CA) coupled to a LTQ-FT mass spectrometer (Thermo Scientific, San Jose, CA) using electrospray ionization. Separation was performed on a Waters Atlantis T3 column (2.1×100 mm, 3 µm) (Waters, Milford, MA) starting with 72 % of solvent A (1 % acetonitrile and 0.1 % formic acid in water) and 28 % of solvent B (1 % water and 0.1 % formic acid in acetonitrile) for 1.5 minutes at a flow rate of 300 µL/min. After 8 minutes the gradient reached 60 % of solvent B. Subsequently, the column was flushed with 100 % B before it was re-equilibrated to initial conditions. The peptide sequences were elucidated using multiple-stage collision-induced dissociation (CID) of the protonated molecule following consecutive cleavages of amino acids residues, starting from the C-terminus of the derivatized linear tetrapeptide. CID was performed with normalized collision energies of 35 %, an isolation width of 1 amu and an activation Q of 0.30.

## Results

### Bioinformatic analysis of a tetrapeptide NRPS

**Figure 2:**
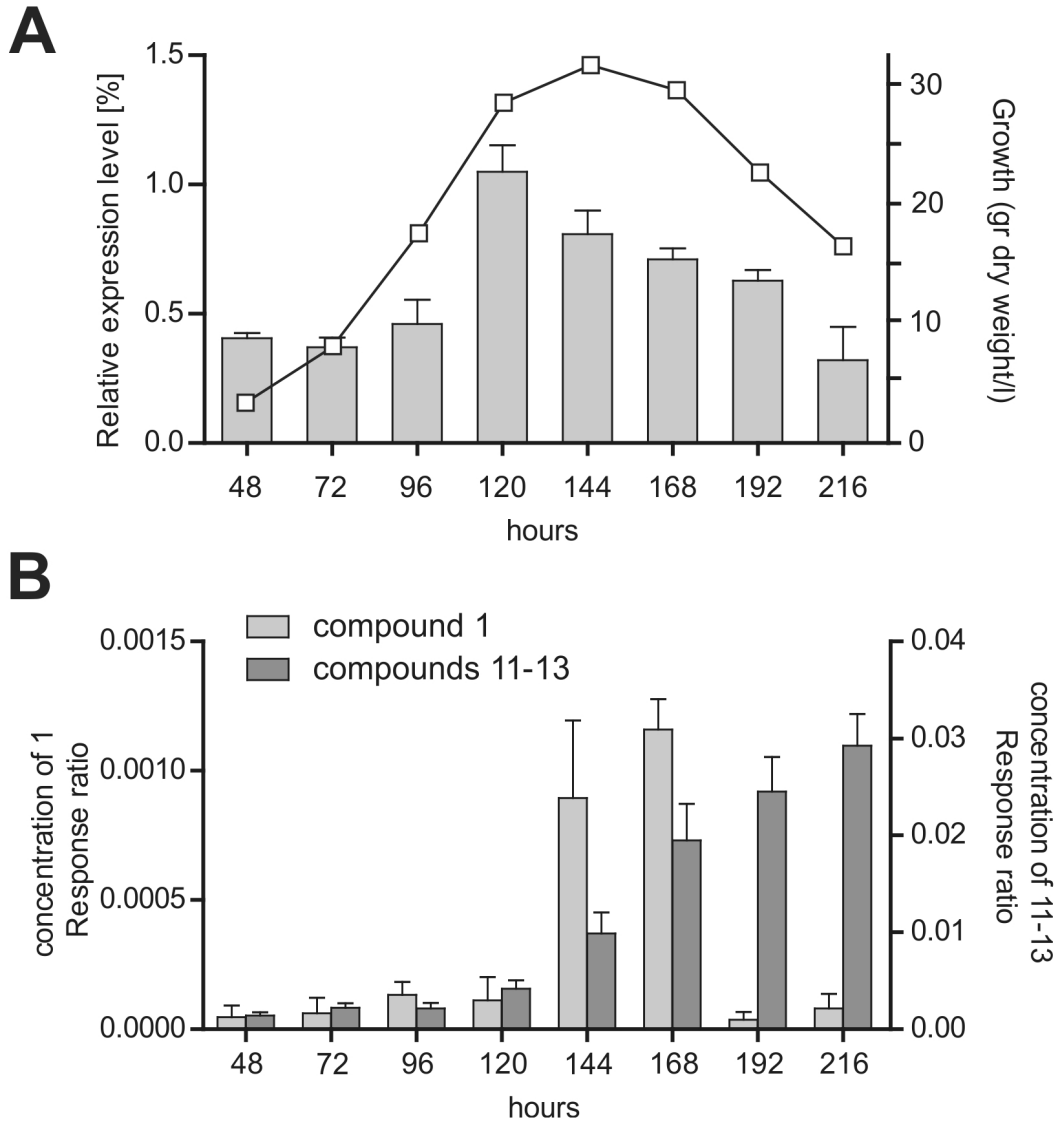
Correlation between the expression level of *HcpA* and metabolite formation during growth. A: Time dependent expression level of *hcpA* as determined by quantitative RT-PCR during growth of *P. chrysogenum* as monitored by biomass formation. B: Internal standard corrected concentration of the cyclic peptide 1 and its degradation products 11–13 present in the growth media. The concentration of peptides was determined by HPLC-UV-MS.

**Figure 3:**
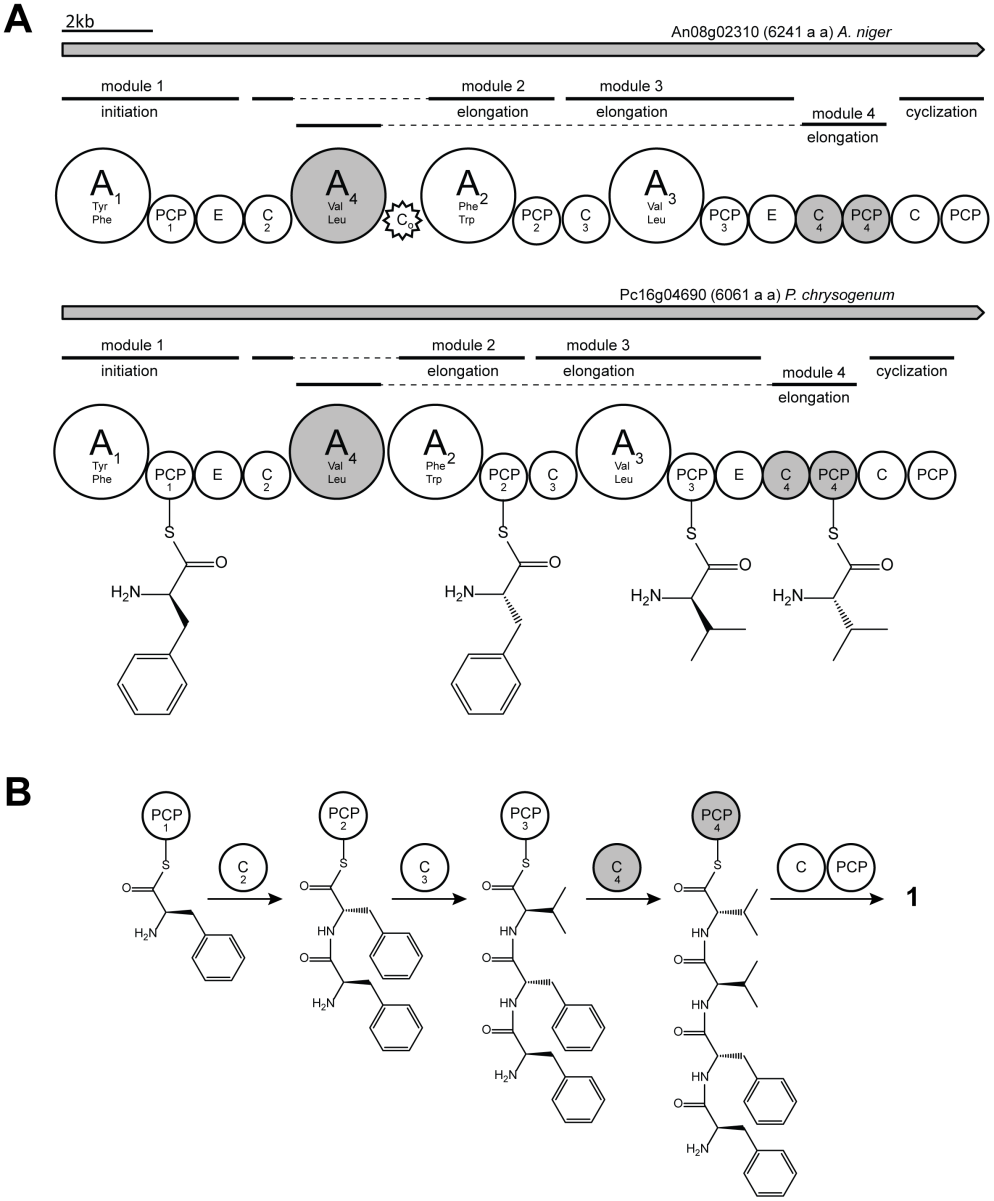
Hypothetical model for the biosynthesis of compound 1 in *P. chrysogenum* and *A. niger* by the HcpA NRPS. A: Binding of the monomers on the carrier protein domains. Both isolated condensation and thiolation domains C_4_ and T_4_, missing a preceding adenylation domain, are assumed to correspond to the adenylation domain A_4_, located upstream. B: Proposed assembly of compound 1 including condensation domains presumed for catalyzing the formation of the peptide bond.

Genome sequencing revealed that *P. chrysogenum* encodes 11 NRPS genes [15]. Microarray expression analysis under glucose-limited chemostat culture conditions as well as quantitative PCR under shake flask culture condition showed that Pc16g04690 (genbank protein identifier CAP93139.1) (*hcpA*) is highly expressed (Figure 2A) [15]. The *hcpA* gene encodes a large multimodular non-ribosomal peptide synthetase enzyme (Figure 3) with 6064 amino acids and a calculated molecular mass of about 670 kDa. HcpA, which shows 54% sequence identity to the orthologous An08g02310 in *A. niger*, has the domain architecture A_1_-PCP_1_-E-C_2_-A_4_-A_2_-PCP_2_-C_3_-A_3_-PCP_3_-E-C_4_-PCP_4_-C-PCP (A =  adenylation, C =  condensation, PCP =  thiolation, and E =  epimerization) (Figure 3A) [15]. A similar domain architecture was deduced for the orthologous protein of *A. niger* except for an insertion of a 177 amino acid long sequence between the adenylation domain A_4_ and A_2_ which shows homology to conserved motifs of an incomplete condensation domain (C_o_) (Figure 3A). To predict the substrate specificity of the four adenylation domains of the two HcpA proteins, NRPSPredictor2 was used [28]. This program extracted the active site amino acid motifs DAACVAGVAK and DAVIIAAVAK as the signature sequences for the A_1_ domain in the *P. chrysogenum* and *A. nidulans* HcpA proteins. This motif exhibits similarity with the signature sequence of the adenylation domain of a bacitracine-producing NRPS that activates phenylalanine as a substrate. The signature sequences for the A_4_ domain (DAVSAGVAAK and DMQSAWFICK in the *P. chrysogenum* and *A. nidulans* HcpA, respectively) shows homology with the valine-activating adenylation domain of the gramicidin synthetase, whereas the A_2_ (DAMTVGGVFK and DVLSTGAICK for the *P. chrysogenum* and *A. nidulans* HcpA, respectively) and A_3_ (DAMFVGGVFK and DAMFVGGIFK for the *P. chrysogenum* and *A. nidulans* HcpA, respectively) domains have predicted specificities towards phenylalanine and valine, respectively. The overall architecture of both synthetases is unusual, as the A_2_ and A_4_ domains occur adjacent to each other, flanked by a single C and PCP domain in a C_2_-A_4_-A_2_-PCP_2_ pattern. On the other hand, an incomplete module (C_4_-PCP_4_) without an adjacent A domain is found at the N-termini of these NRPSs (Figure 3A).

### Genetic deletion of the tetrapeptide NRPS and secondary metabolite identification

**Figure 4:**
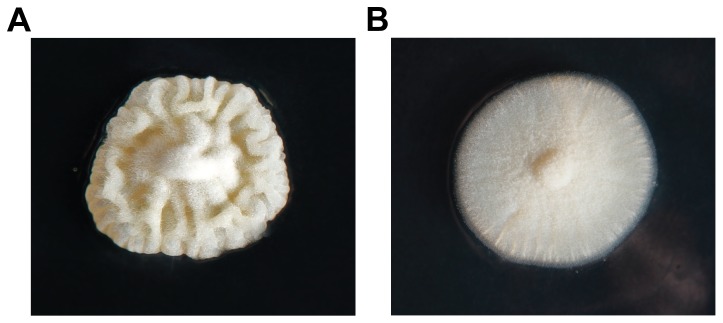
Colonies of *P. chrysogenum* strain DS54555. A: Colony of the wild type strain showing a wrinkled surface. B: Colony of the *ΔhcpA* strain with a smooth surface.

**Figure 5:**
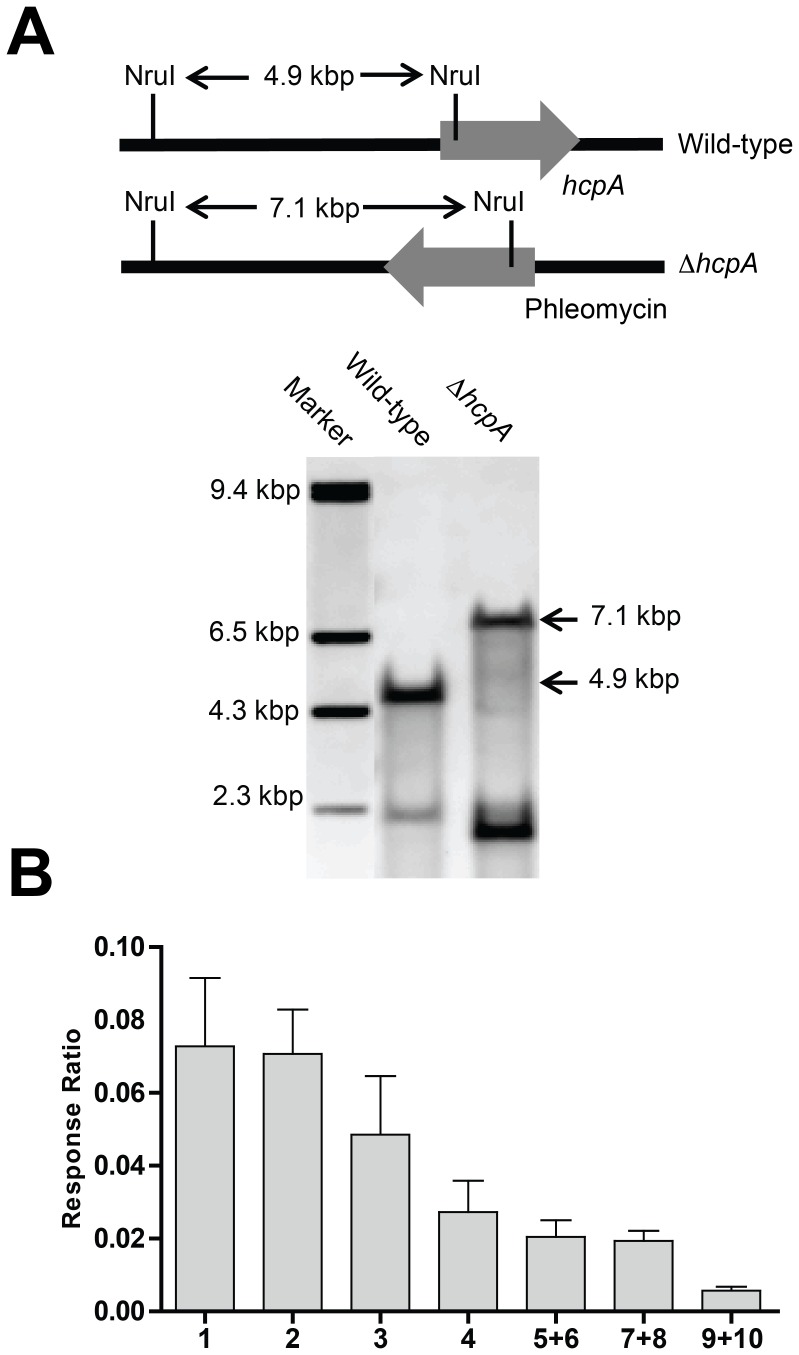
Southern blot analysis for the *hcpA* deletion and concentration of cyclic tetrapeptides in culture broth of host strain. A: Southern blot hybridization validating the complete gene deletion of *hcpA.* B: Internal standard corrected concentrations of the cyclic tetrapeptides 1–10 present in the culture broth of the host strain of *P. chrysogenum* grown for 168 hours. Isomers are presented together as no chromatographic separation was obtained during profiling. No cyclic or linear tetrapeptides could be found in the deletion strain.

In order to identify the secondary metabolites synthetized by HcpA, the corresponding gene was deleted and comparative metabolite profiling was performed on the culture supernatant of the host and deletion strain. As host strain, *P. chrysogenum* DS54555 was used, which is derived from the industrial DS17690 strain and that lacks the *ku70* gene to make it competent for homologous recombination. The DS54555 strain also lacks the multiple penicillin biosynthetic genes clusters in order to facilitate the detection of unknown secondary metabolites in the culture broth as the profile is no longer dominated by β-lactams. The *hcpA* gene was resequenced from the genome of *P. chrysogenum* DS54555 and the nucleotide sequence of the open reading frame (genbank KJ679502) including the promotor region was found to be identical to the *hcpA* gene present in the sequenced genome of *P. chrysogenum* Wisconsin54-1255 [15]. The gene deleted by homologous recombination using the deletion plasmid pDEST R_4_-R_3_p (Figure S1) containing the flanking regions of *hcpA* and the phleomycin resistance gene. Colonies were selected on phleomycin containing agar plates where the mutant colonies showed smooth phenotypic characteristics compared to the wrinkled surface of the colonies of the parental strain (Figure 4). The deletion of the *hcpA* gene was confirmed by southern blot hybridization (Figure 5A).

**Figure 6:**
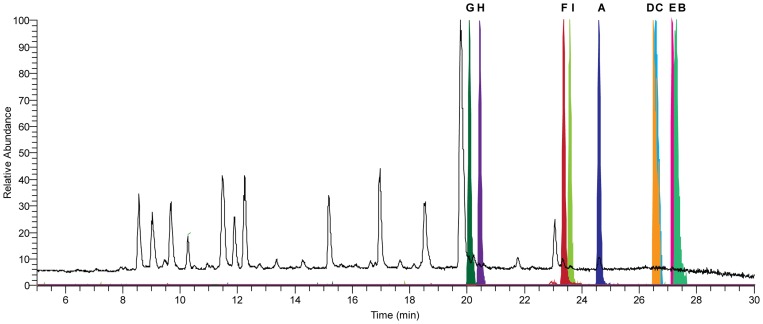
HPLC-MS elution profiles of the internal standard reserpine and the highest abundant identified peptides. Total ion chromatogram (TIC, black) and normalized extracted ion chromatograms (EIC, colored) of the protonated molecule ([M+H]^+^ with 5 ppm accuracy) of the used internal standard reserpine (A), the four highest abundant cyclic tetrapeptides 1 (B), 2 (C), 3 (D), 4 (E) and linear tetrapeptides 11–13 (F), 14–16 (G), 17–19 (H), 20–22 (I) present in the culture broth of *P. chrysogenum*.

The host and *ΔhcpA* strain were grown for 168 hours in SMP Medium followed by comparative metabolite analysis using HPLC-UV-MS. Several secondary metabolites were found to be present in the host but absent in the deletion strain (Figure 6, Table S3). These compounds could be classified into two groups according their chemical structure. The first group consists of ten cyclic tetrapeptides, which were identified using HPLC-MS^n^, NMR and a synthetic standard (Figure 1). Upon excitation, cyclic tetrapeptides undergo ring opening in the mass spectrometer resulting in four linear tetrapeptides, which can be sequenced in a similar fashion as linear peptides. By following the sequential loss of amino acids from b-ions of each generated linear tetrapeptide, the sequence of their cyclic origin could be determined (Figure S2). The identities of the involved amino acids, corresponding to the losses in the mass spectrometer, as well as their sequence were additionally confirmed by ^1^H-NMR and ^13^C-NMR experiments for the compounds **1** and **2** (Table S4 and S5). To discriminate the amino acid isoleucine from its isomer leucine, which is represented by a loss of 113 Da (C_6_H_11_ON) in the mass spectra of compounds **5–10**, ^1^H-, ^13^C- and various 2D-NMR experiments were conducted (Figure S3, Table S6). Overall, cyclic tetrapeptides obtained from metabolic profiling contain various combinations of the five amino acids valine, isoleucine, phenylalanine, tyrosine and tryptophan making them extremely hydrophobic. They are arranged in a common sequence in which two aromatic amino acids are followed by two aliphatic amino acids. The absolute stereochemistry of compound **2** was confirmed by spiking its synthetic standard to a natural extract, which did not lead to additional signals in the ^1^H-NMR spectra, whereas the intensity of the main signals increased as compared to the impurities (Figure S4A and B). In addition, superimposing the HMBC spectra of the natural and the synthetic sample of peptide **2** illustrated identical correlations and shifts (Figure S5). Furthermore, retention time and MS^2^ fragmentation did not show differences between the extracted compound and the synthesized standard. This leads to the conclusion that not only the sequence of amino acids is identical in the synthetic and natural peptide, but also the chirality of the individual amino acids. Therefore, the cyclic tetrapeptide **2** has the same stereochemistry as observed for **1** [19] with the first amino acid of an aliphatic and aromatic pair in D- and the second amino acid in L-form. Due to the low concentration of cyclic tetrapeptides **3–10**, reliable stereochemical information could not be obtained. However, as these peptides originate from the same NRPS as **1–2** and share the same sequence of aliphatic and aromatic amino acids, identical stereochemistry is expected.

The second category of identified compounds consists of 18 linear tetrapeptides which are comprised of the same five amino acids as their cyclic analogues (Table S3 and S7). Similar to the cyclic peptides, each linear tetrapeptide contains two aliphatic and two aromatic amino acids in different arrangements yielding various isomeric structures. Due to similar chromatographic properties and an identical mass-over-charge ratio, these isomers are represented as a group in data obtained from metabolic profiling (Figure S6A and B). Their structure elucidation is challenging as minor fragments can be attributed to a fragmentation of low abundant linear tetrapeptides as well as to possible sequence scrambling of major linear tetrapeptides which was reported for similar linear peptides [29]. To separate and sequence these isomeric tetrapeptides and to prevent possible sequence scrambling, their N-terminus was derivatized using 6-aminoquinolyl-N-hydroxysuccinimidyl carbamate (AQC) (Figure S6C and D) [30]. De novo peptide sequencing was performed by following the consecutive amino acid losses of various b-ions using multiple-stage fragmentation mass spectrometry (Figure S6E and Table S7). Similar to the cyclic peptides, each linear tetrapeptide incorporates two consecutive aromatic and/or two consecutive aliphatic amino acids.

In conclusion, several linear and cyclic tetrapeptides with similar structural features were found to be present in the host but absent in the deletion strain which shows that they originate from one single NRPS, namely HcpA.

### Expression of the *hcpA* gene and secondary metabolite production

To examine the expression of the *hcpA* gene, the host strain was grown for up to 216 hours in SMP medium. Samples were collected for total mRNA extraction and extracellular metabolites analysis during growth. Metabolite concentrations were determined by HPLC-UV-MS, while transcript levels were determined by quantitative PCR using γ-actin as a reference gene. A high expression level of the *hcpA* gene was observed between 120 and 168 hours of growth, which was paralleled by a 12 times increase in the concentration of cyclic tetrapeptide **1** (Figure 2A and B) in the medium. In general, the concentration of cyclic tetrapeptides was exceptionally high around 168 hours of growth, while the concentration of the linear tetrapeptides increased with time. These data suggest that the linear tetrapeptides are derived from the cyclic tetrapeptides by degradation.

## Discussion

Here we have demonstrated that the NRPS HcpA is responsible for the biosynthesis of cyclic hydrophobic tetrapeptides. Bioinformatics analysis of HcpA shows an unusual domain architecture in comparison to classical linear NRPSs with no products associated so far. However, through the deletion of the *hcpA* gene and comparative metabolite profiling, ten cyclic tetrapeptides were identified, including the previously described secondary metabolite fungisporin **1** [19]. According to the non-ribosomal code in combination with discovered cyclic products, the first NRPS module is specific for phenylalanine. Due to the adjacent E domain, responsible for epimerization of the activated amino acid, a D-configuration is expected as observed for product **1** (Figure 1). The next two modules of the NRPS contain an unusual architecture in which PCP_2_ and C_3_ are flanked by two neighboring adenylation domains A_2_ and A_4_. As adenylation domain A_4_ shows high specificity towards valine and adenylation domain A_2_ shows high homology towards A_1_, both adenylation domains activate phenylalanine as supported by the structure of **1**. Due to the lack of an adjacent PCP domain for A_4_ and a missing C domain for A_2_, which are necessary for loading and condensation of the substrate, no module activity is expected according to the classical C-A-PCP geometry (Figure 3A). Surprisingly, both products **1** and **2** show the incorporation of L-phenylalanine at the second position. As module 2 is predicted to be the only module capable to catalyze the incorporation of L-phenylalanine, A_2_ indeed must be active. Consequently, it seems likely that A_4_ is skipped, leaving the N-terminal C_2_ domain of module 2 to catalyze the condensation of the first amino acid from module 1 to the second amino acid from module 2, as observed for the products **1** and **2**. The third module of HcpA contains the domains C_3_, A_3_, PCP_3_ and E arranged in a common linear order. The A_3_ domain is predicted to activate valine, which agrees with the peptide sequence of **1** and **2**, as D-valine is their third amino acid. The fourth module of the NRPS is an incomplete module consisting of C_4_ and PCP_4_. Due to a missing preceding A domain no activity is expected. However, the chemical structures of both cyclic tetrapeptides **1** and **2** show the incorporation of L-valine as fourth amino acid in their peptide sequence. As A_4_ is the only domain predicted to be specific to valine without an adjacent epimerization domain, it is very likely that this domain is a ‘trans-acting’ A domain that interacts with C_4_ and PCP_4_ to add the last amino acid to the tetrapeptide. A similar architectural flexibility has been observed in the biosynthesis of yersiniabactin, in which one A domain, located in HMWP2, loads three PCPs located on different modules [31]–[33]. As the linear domain organization of HcpA does not reflect a linear assembly of substrate incorporation into the final product, non-linear interactions are deduced. Although A_4_, C_4_ and PCP_4_ are not in a consecutive sequence on a genomic level, they might still be closely arranged in the final three-dimensional enzymatic structure. Structural characterization would be necessary to determine spatial proximity. Finally, after the incorporation of L-valine into the peptide chain, the C and PCP domain of the last module catalyze the cyclization of the peptide leading to the final cyclic structure, as previously observed in other NRPS systems [34], [35]. It should be stressed that non-linear NRPS organizations are a very heterogenous group of NRPS systems which deviate from the colinearity rule thus showing various unusual mechanisms [6]. In an alternative model A4 might be non-functional, leaving A3 loading two PCPs, namely PCP3 and PCP4, similar to the cysteine-specific A domain of HMWP2 in yersiniabactin biosynthesis [3]. However, more detailed biochemical studies are required to fully understand the interplay between these enzymes and to confirm the exact biosynthetic mechanisms involved.

Next to the production of **1** and **2**, eight additional lower abundant cyclic tetrapeptides were identified to be present in the host strain and absent in the deletion strain (Figure 1). They show a similar peptide sequence as **1**, containing two aromatic amino acids followed by two aliphatic amino acids. Although stereochemical information is only available for the compounds **1** and **2**, it can be assumed that each of the cyclic tetrapeptides contains an aliphatic and aromatic amino acid in the D configuration, more specifically at the first and third position. These assumptions in combination with the stereochemical structure of **1** and **2** lead to the conclusion, that each adenylation domain of HcpA shows specificity towards more than one precursor amino acid with A_1_ being specific towards phenylalanine and tyrosine and A_2_ being specific towards phenylalanine and to a lesser extent to tryptophan (Table S8) reminiscent of microheterogenicity. Together with the two aliphatic amino acid selecting adenylation domains A_3_ and A_4_, which preferably activate valine before isoleucine, 16 cyclic tetrapeptide combinations are theoretically possible. However, only ten of these were detected in the fermentation broth of *P. chrysogenum* confirming a different degree of specificity towards their precursors. Based on a similar chemical scaffold of identified compounds **1–10** to the tetrapeptides cyclo-(*N*-MePhe-Ile)_2_, cyclo-(*N*-MePhe-Val)_2_ and cyclo-(*N*-MePhe-Val-*N*-MePhe-Ile) reported from *Onychocola sclerotic*, cardiac channel blocking activities can be expected for the hydrophobic cyclic peptides presented here [36]. In addition, the colonies of the Δ*hcpA* strain lost the ability to produce a wrinkled surface leading to a rather smooth appearance (Figure 4). As this change is attributed to the deletion of the *hcpA* gene, the hydrophobic cyclic peptides **1–10** need to be involved. Possibly, these molecules function analogous to hydrophobins in altering the surface properties and influencing aerial growth. The exact function is, however, still unclear.

In addition to the cyclic tetrapeptides several highly abundant linear tetrapeptides could be observed in the cultural broth of the host strain that were absent in the deletion strain. To each of the cyclic tetrapeptides, several linear tetrapeptides with the same sequence were present. For instance, for the cyclic tetrapeptide **1** with the sequence *cyclo-*(Phe-Phe-Val-Val), three linear tetrapeptides with the sequences Phe-Val-Val-Phe, Val-Phe-Phe-Val and Phe-Phe-Val-Val could be found at different ratios. Their concentration increased over time in the media while their cyclic counterpart decreased after 168 hours (Figure 2B). This leads to the conclusion that the linear peptides originate from the degradation of their cyclic counterparts by hydrolysis of their peptide bonds, which was observed exclusively between two aromatic, two aliphatic or an aliphatic followed by an aromatic amino acid (Table S3 and S7). Linear tetrapeptides with a N-terminal aliphatic amino acid and a C-terminal aromatic amino acid were not detected, leading to the conclusion that cleavage of this bond is not favorable. As cyclic tetrapeptides are relatively stable towards chemical and thermal degradation, enzymatic hydrolysis might be most probable.

*P. chrysogenum* contains a second NRPS that could potentially be involved in tetrapeptide formation, i.e., Pc13g14330. This protein has a linear organization and thus differs from the HcpA protein. Pc13g14330 is hardly expressed under batch culture conditions as employed in this study. Moreover, overexpression of Pc13g14330 under control of the strong *pcbC* promoter did not lead to any novel detectable metabolite in the growth medium, nor did the deletion of the Pc13g14330 gene affect the cyclic tetrapeptide production or result in a loss of other metabolites (unpublished data). Therefore, we conclude that Pc13g14330 is unrelated to HcpA, and not responsible for cyclic tetrapeptide formation. HcpA shows 54% amino acid sequence identity with the orthologous protein from *A. niger* with exactly the same module organization (Figure 3A). Furthermore, all cyclic products **1–10** present in *P. chrysogenum* could also be found in the supernatant of an *A. niger* strain while they were absent in the *HcpA* deletion strain (unpublished data). Therefore, it is concluded that HcpA is involved in production of all cyclic metabolites (**1–10**) in *A. niger*. A small difference exists in the organization of both HcpA proteins with a short additional amino acid sequence present between domains A_4_ and A_2_ in the *A. nidulans* enzyme. This sequence showed limited homology to a condensation domain and appears as an incomplete condensation domain. Hence, one may deduce that this non-canonical situation might have evolved quite recently. Perhaps, a complete C-A-PCP-C-A-PCP module structure was present before, but degenerated after new interactions between the domains evolved.

## Conclusions

Cyclic tetrapeptides are a structurally interesting group of peptides produced by fungi that have attracted much interest because of their cardiac ion-channel blocking properties. The complete biosynthetic mechanism of these metabolites has been deduced by gene deletion experiments in *P. chrysogenum* in combination with comparative metabolite profiling and consecutive structure elucidation. By this analysis, *trans*-aminoacylation for chain elongation in NRPS has been found. Furthermore, a distinct microheterogenicity of each adenylation domain towards different amino acid building blocks resulted in a range of cyclic tetrapeptides as produced by a single NRP synthetase.

## Supporting Information

Figure S1 **Plasmid used for the deletion of the** ***hcpA*** **gene in** ***P. chrysogenum*** **.**
(TIF)

Figure S2 **Multiple-stage fragmentation for de novo sequencing of compound 2 based on sequential amino acid losses.** A: MS^2^ fragmentation spectra of the cyclic tetrapeptide 2 *cyclo*-(*d*-Tyr-*l*-Phe-*d*-Val-*l*-Val). Due to ring opening of the cyclic peptide in the mass spectrometer at different positions, three different amino acid losses occurred, yielding different b_3_-ions. B-D: MS^3^ fragmentation spectra obtained by further fragmenting b_3_-ions from MS^2^ showing b_2_-ions used for peptide sequencing. The cyclic tetrapeptides 1 and 3–10 were identified accordingly.
(TIF)

Figure S3 **HSQC spectrum of isolated mixture of cyclic tetrapeptides in DMSO.** The spectra were used for the identification of isoleucine present in the minor abundant products 5 and 9. Signals corresponding to isoleucine are indicated with circles. Conducted TOCSY, COSY and HMBC experiments further confirm this conclusion (data not shown). ^1^H and ^13^C chemical shifts are shown in Table S6.
(TIF)

Figure S4 **^1^H-NMR spectra of mixtures of cyclic tetrapeptides.** A: ^1^H-NMR spectrum of a precipitate of various cyclic tetrapeptides containing primarily peptide 1 and 2 (bottom). Synthetic compound 2 spiked to the precipitated mix of various cyclic tetrapeptides in DMSO at 340 K (top). Signals corresponding to 2 were increased as compared to the impurities whereas additional signals did not appear. B: Zoomed regions of ^1^H-NMR spectrum of natural precipitate (bottom) and precipitate spiked with compound 2 (top). Signals which increased after spiking are indicated (*).
(TIF)

Figure S5 **Superimposed HMBC spectra of synthetic compound 2 (red) and an isolated mixture of cyclic tetrapeptides containing naturally produced compound 2 (black).** Correlations between NH and CO are shown which indicate identical shifts for both samples. Assignments can be found in Table S4.
(TIF)

Figure S6 **Sequencing of the linear isomers 11 (Phe-Phe-Val-Val), 12 (Val-Phe-Phe-Val) and 13 (Phe-Val-Val-Phe).** A: Extracted ion chromatogram (EIC) of the linear tetrapeptides 11, 12 and 13 using the profiling method. No chromatographic separation could be achieved. B: MS^2^ fragmentation spectrum of unseparated linear tetrapeptides 11–13. Although the spectrum is dominated by fragments originating from 11, several lower abundant fragments can be found originating from a fragmentation of 12 and 13 or possible sequence scrambling of 11. C: Sequence of linear tetrapeptides after N-terminal AQC derivatization to achieve better chromatographic separation and to prevent sequence scrambling. D: Normalized total ion chromatogram (TIC) of AQC derivatized linear peptides 11–13 after first C-terminal amino acid loss showing chromatographic separation and allowing peptide sequencing. E: Individual MS^3^ fragmentation spectra of chromatographically separated derivatized peptides 11, 12 and 13 showing b_2_ and b_1_ ions used for peptide sequencing. The linear peptides 14–28 were identified accordingly.
(TIF)

Table S1 **Primers designed for making the** ***hcpA*** **deletion construct in** ***P. chrysogenum*** **.**
(DOCX)

Table S2 **Primers designed for the expression analysis of the** ***hcpA*** **and actin gene.**
(DOCX)

Table S3 **Retention time, formula and acquired m/z of cyclic and linear tetrapeptides obtained from metabolic profiling.** Isomers have the same retention time as they could not be chromatographically separated during profiling.
(DOCX)

Table S4 **^1^H and ^13^C-NMR chemical shifts of synthetically produced compound 2 with sequence** ***cyclo*** **-(** ***d*** **-Tyr-** ***l*** **-Phe-** ***d*** **-Val-** ***l*** **-Val) in DMSO at 340 K.** As synthetically and naturally produced **2** show identical NMR spectra, only chemical shifts for the synthetically produced compound are shown. δ_DMSO_ (^1^H/^13^C)  =  (2.55/40.50), (δ in ppm).
(DOCX)

Table S5 **^1^H and ^13^C chemical shifts of naturally produced compound 1 with sequence** ***cyclo*** **-(** ***d*** **-Phe-** ***l*** **-Phe-** ***d*** **-Val-** ***l*** **-Val) present in a mix of various cyclic tetrapeptides in DMSO acquired at 340 K.** Signals overlapping with the highest abundant compound **2** are indicated (*). δ_DMSO_ (^1^H/^13^C)  =  (2.55/40.50), (δ in ppm).
(DOCX)

Table S6 **^1^H and ^13^C chemical shifts of isoleucine in compound 5 with sequence** ***cyclo-*** **(Phe-Phe-Val-Ile) and compound 9 with sequence** ***cyclo-*** **(Phe-Phe-Val-Ile) present in a extracted mix of cyclic tetrapeptides.** NMR signals corresponding to C = O and NH as well as remaining amino acid signals are not observed (n.o.) due to overlap with the main constituents. δ_DMSO_ (^1^H/^13^C)  =  (2.55/40.50).
(DOCX)

Table S7 **Sequencing data leading to the identification of linear tetrapeptides after AQC derivatization.** Multiple-stage fragmentation was used for determination of b-ions for de-novo peptide sequencing.
(DOCX)

Table S8 **Identified and proposed cyclic and linear tetrapeptides in respect to the adenylation domain specificity in the NRPS HcpA.**
(DOCX)
