# Supplementary figures and images for: A Non-Canonical NRPS Is Involved in the Synthesis of Fungisporin and Related Hydrophobic Cyclic Tetrapeptides in Penicillium chrysogenum
Source: PLoS One. 2014 Jun 2;9(6):e98212. doi: 10.1371/journal.pone.0098212 (PMC4041764; doi:10.1371/journal.pone.0098212)

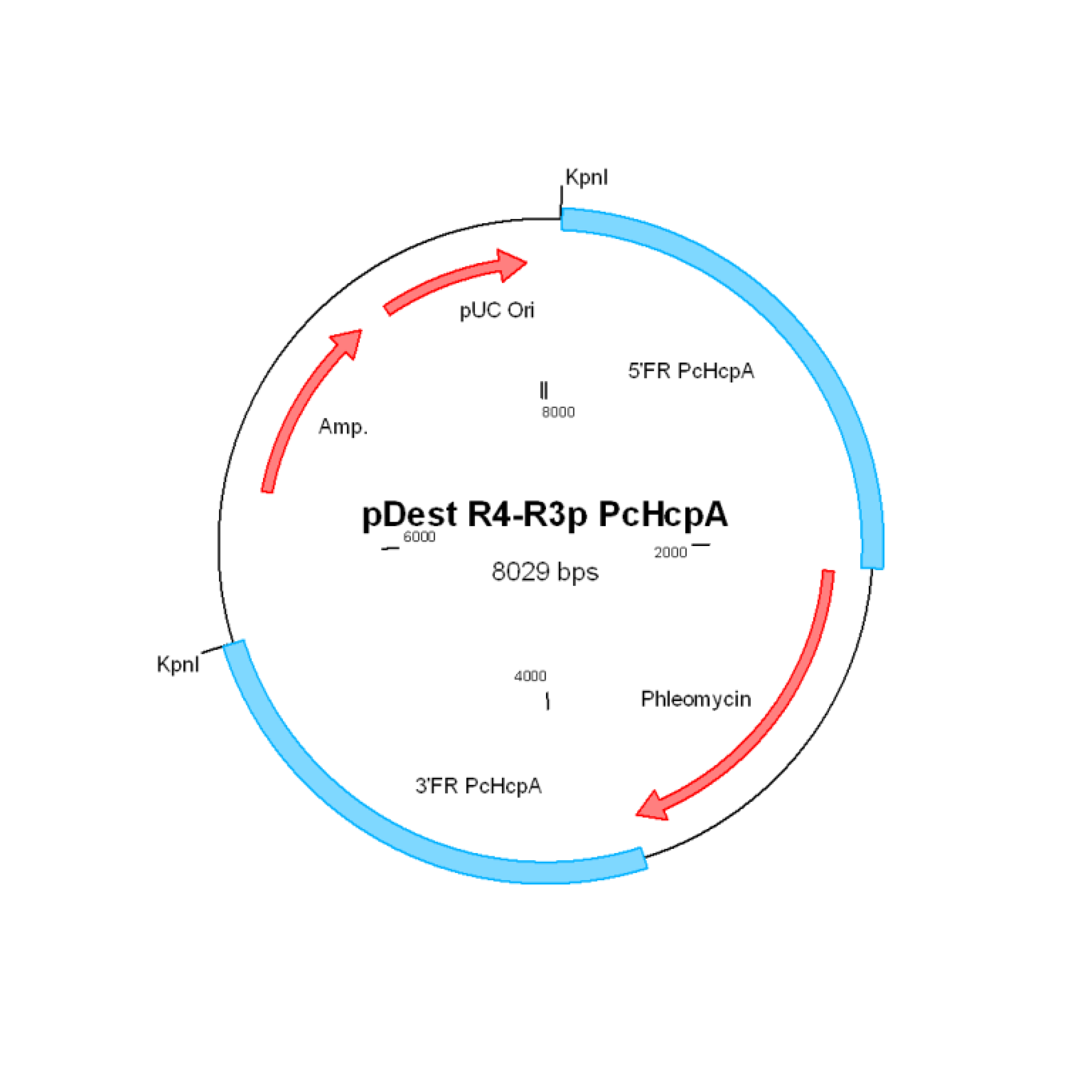

Supplement: Figure S1 — Plasmid used for the deletion of the hcpA gene in P. chrysogenum . (TIF) [file pone.0098212.s001.tif]

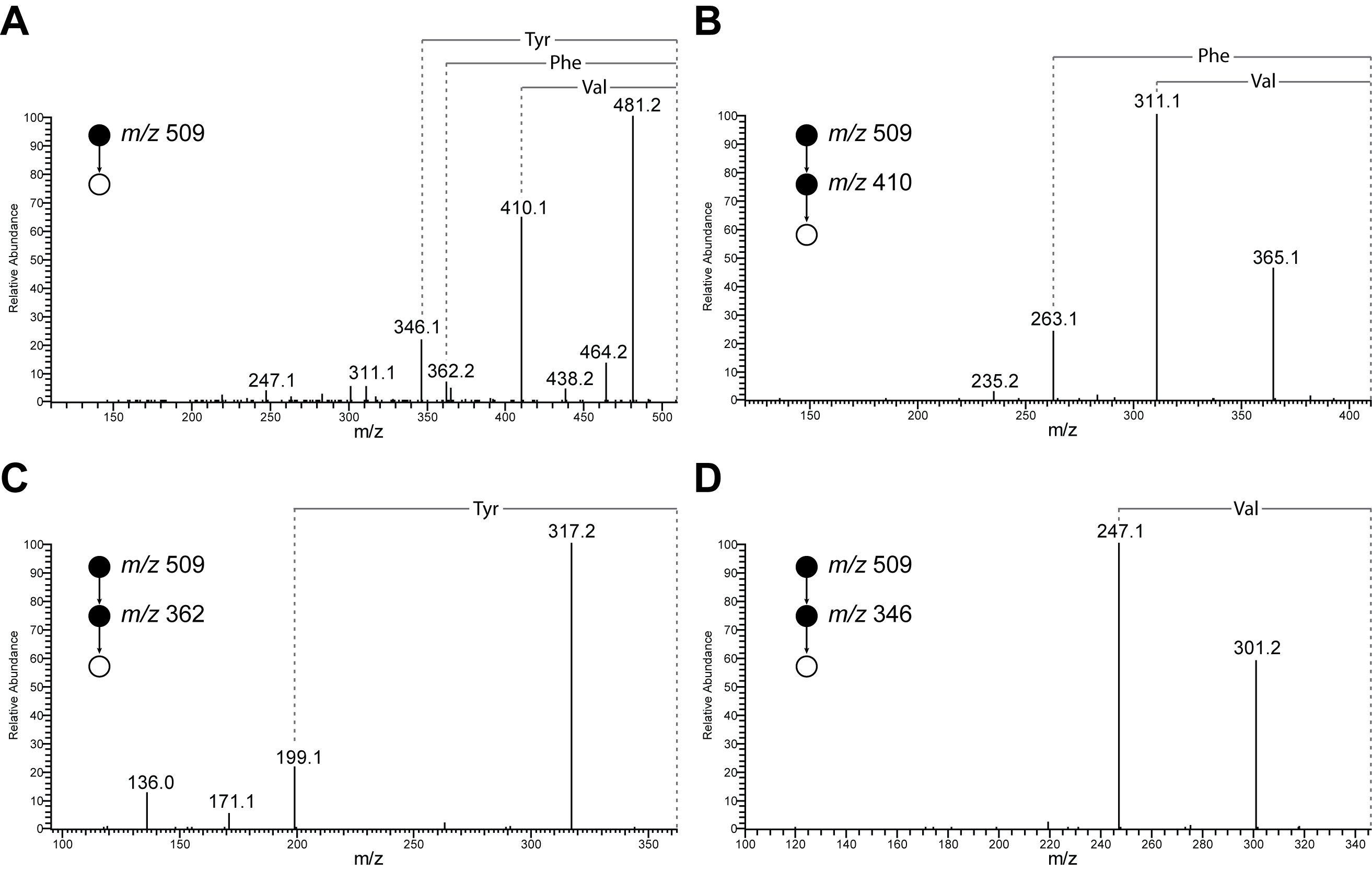

Supplement: Figure S2 — Multiple-stage fragmentation for de novo sequencing of compound 2 based on sequential amino acid losses. A: MS2 fragmentation spectra of the cyclic tetrapeptide 2 cyclo-(d-Tyr-l-Phe-d-Val-l-Val). Due to ring opening of the cyclic peptide in the mass spectrometer at different positions, three different amino acid losses occurred, yielding different b3-ions. B-D: MS3 fragmentation spectra obtained by further fragmenting b3-ions from MS2 showing b2-ions used for peptide sequencing. The cyclic tetrapeptides 1 and 3–10 were identified accordingly. (TIF) [file pone.0098212.s002.tif]

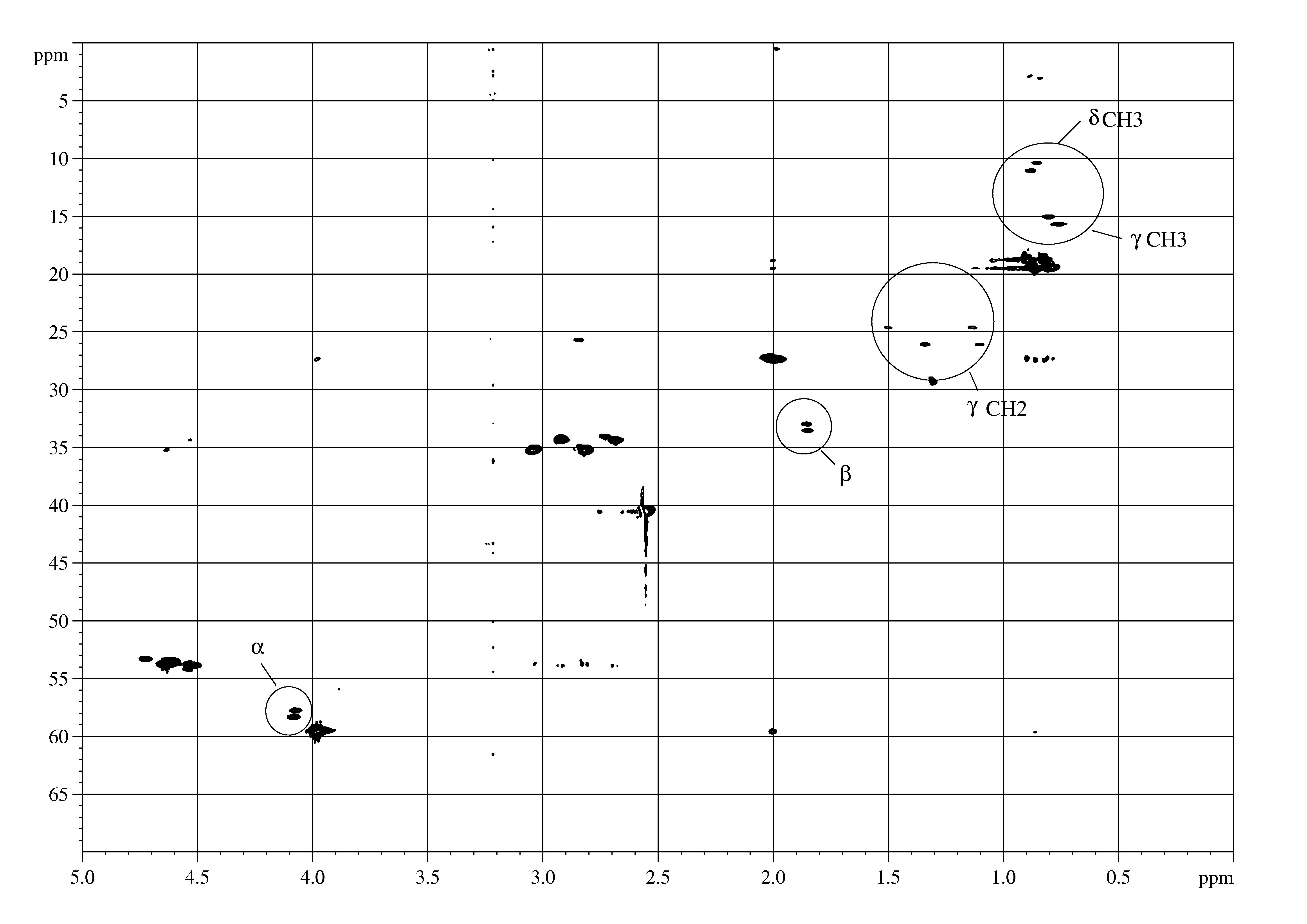

Supplement: Figure S3 — HSQC spectrum of isolated mixture of cyclic tetrapeptides in DMSO. The spectra were used for the identification of isoleucine present in the minor abundant products 5 and 9. Signals corresponding to isoleucine are indicated with circles. Conducted TOCSY, COSY and HMBC experiments further confirm this conclusion (data not shown). 1H and 13C chemical shifts are shown in Table S6. (TIF) [file pone.0098212.s003.tif]

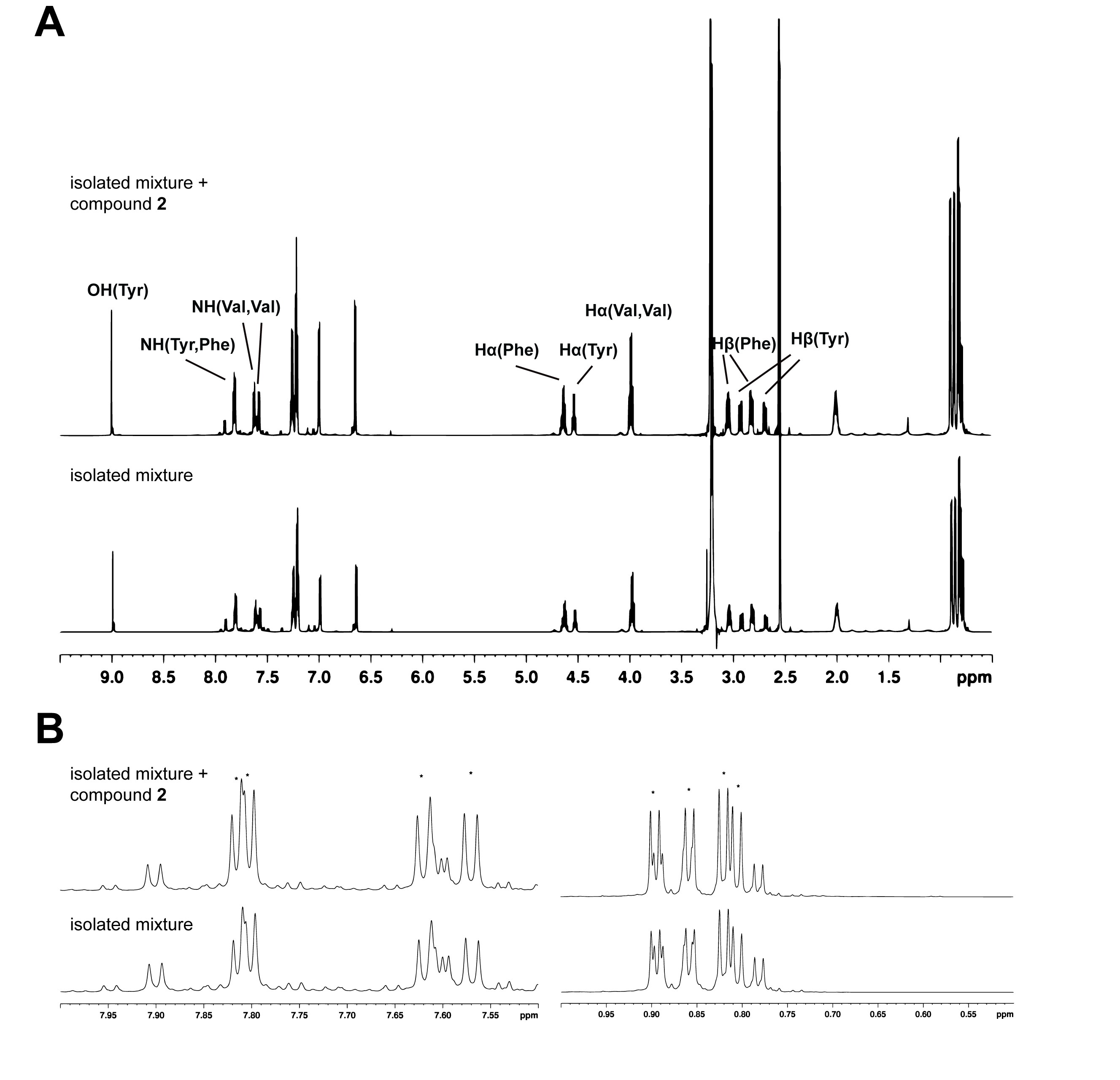

Supplement: Figure S4 — 1H-NMR spectra of mixtures of cyclic tetrapeptides. A: 1H-NMR spectrum of a precipitate of various cyclic tetrapeptides containing primarily peptide 1 and 2 (bottom). Synthetic compound 2 spiked to the precipitated mix of various cyclic tetrapeptides in DMSO at 340 K (top). Signals corresponding to 2 were increased as compared to the impurities whereas additional signals did not appear. B: Zoomed regions of 1H-NMR spectrum of natural precipitate (bottom) and precipitate spiked with compound 2 (top). Signals which increased after spiking are indicated (*). (TIF) [file pone.0098212.s004.tif]

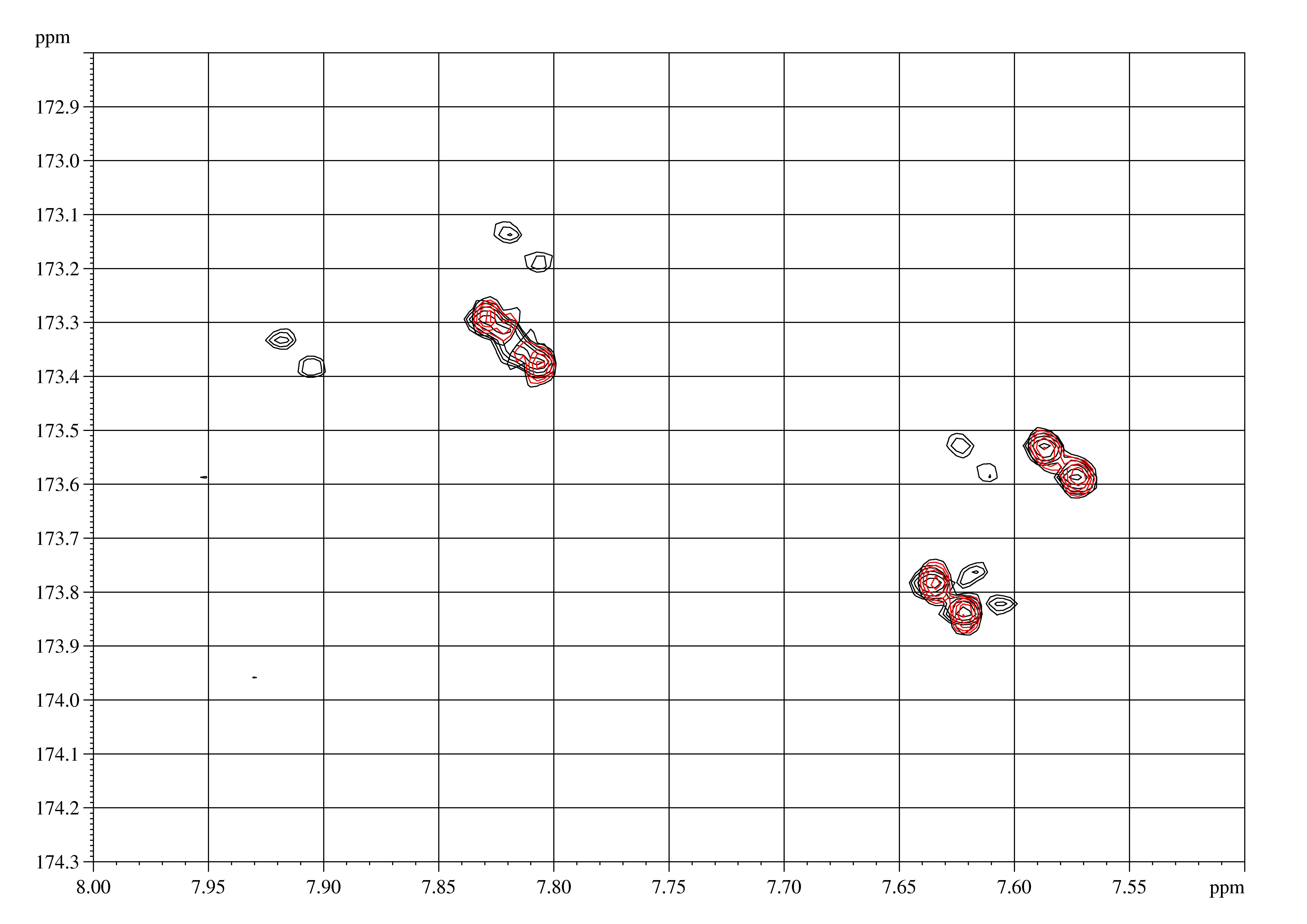

Supplement: Figure S5 — Superimposed HMBC spectra of synthetic compound 2 (red) and an isolated mixture of cyclic tetrapeptides containing naturally produced compound 2 (black). Correlations between NH and CO are shown which indicate identical shifts for both samples. Assignments can be found in Table S4. (TIF) [file pone.0098212.s005.tif]

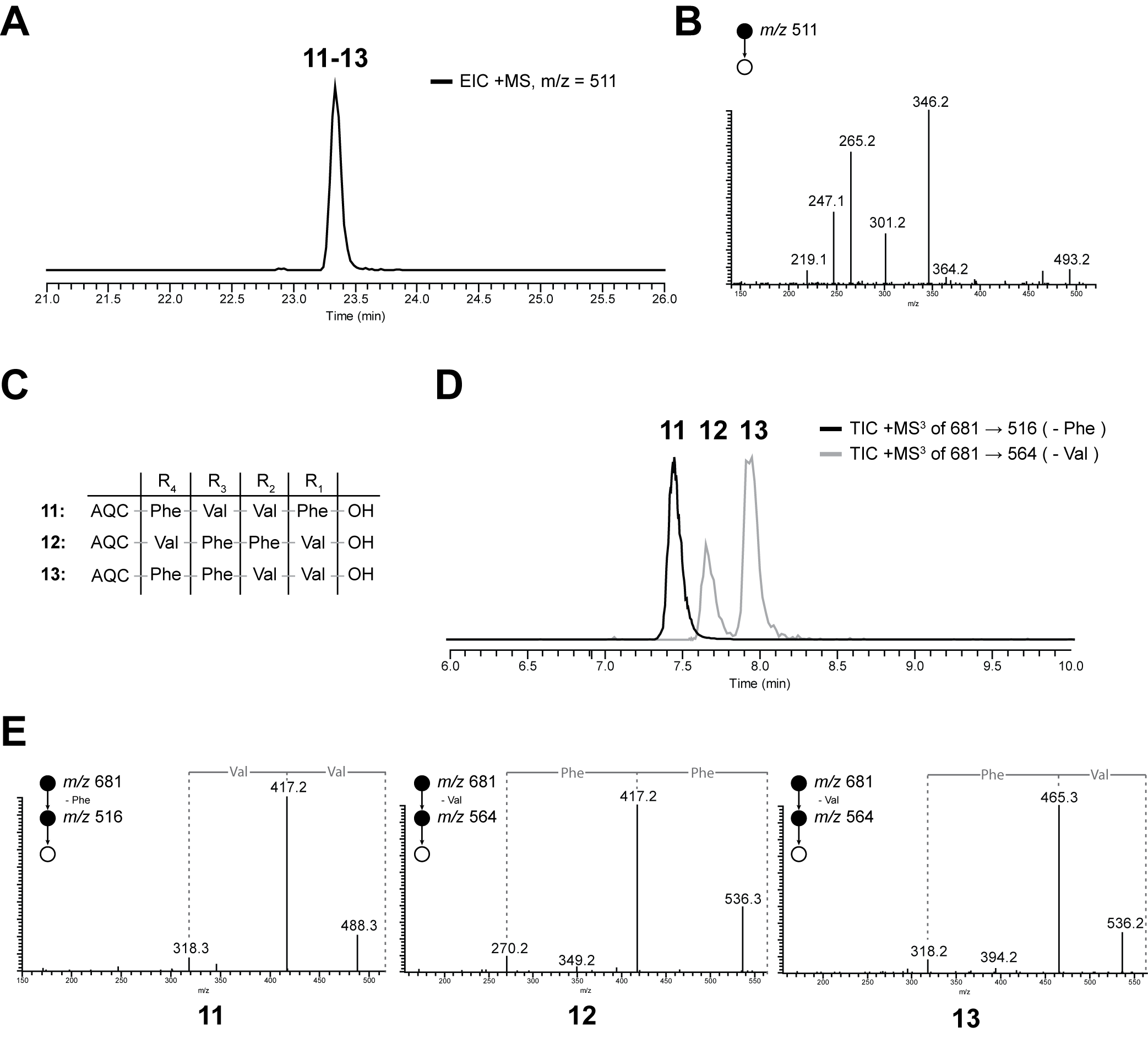

Supplement: Figure S6 — Sequencing of the linear isomers 11 (Phe-Phe-Val-Val), 12 (Val-Phe-Phe-Val) and 13 (Phe-Val-Val-Phe). A: Extracted ion chromatogram (EIC) of the linear tetrapeptides 11, 12 and 13 using the profiling method. No chromatographic separation could be achieved. B: MS2 fragmentation spectrum of unseparated linear tetrapeptides 11–13. Although the spectrum is dominated by fragments originating from 11, several lower abundant fragments can be found originating from a fragmentation of 12 and 13 or possible sequence scrambling of 11. C: Sequence of linear tetrapeptides after N-terminal AQC derivatization to achieve better chromatographic separation and to prevent sequence scrambling. D: Normalized total ion chromatogram (TIC) of AQC derivatized linear peptides 11–13 after first C-terminal amino acid loss showing chromatographic separation and allowing peptide sequencing. E: Individual MS3 fragmentation spectra of chromatographically separated derivatized peptides 11, 12 and 13 showing b2 and b1 ions used for peptide sequencing. The linear peptides 14–28 were identified accordingly. (TIF) [file pone.0098212.s006.tif]
