# Supplementary material for: A Non-Canonical NRPS Is Involved in the Synthesis of Fungisporin and Related Hydrophobic Cyclic Tetrapeptides in Penicillium chrysogenum
Source: PLoS One. 2014 Jun 2;9(6):e98212. doi: 10.1371/journal.pone.0098212 (PMC4041764; doi:10.1371/journal.pone.0098212)
Supplement: Table S1 — Primers designed for making the hcpA deletion construct in P. chrysogenum . (DOCX) [file pone.0098212.s007.docx]

| No. | Primer sequence (5’ – 3’) |
| --- | --- |
| *attB4F* | GGGGACAACTTTGTATAGAAAAGTTGGGTACCACAACGCCGAACTGCGGGCGC |
| *attB1R* | GGGGACTGCTTTTTTGTACAAACTTGGTGGTTCAAGTGCGCCCGCG |
| *attB2F* | GGGGACAGCTTTCTTGTACAAAGTGGCCATGATCAAGGTCGAATCCGCG |
| *attB3R* | GGGGACAACTTTGTATAATAAAGTTGGGTACCAATTGCAGGCTCGACATGGGCC |

**Table S1. Primers designed for making the *hcpA* deletion construct in *P. chrysogenum*.**
