# Supplementary material for: A Non-Canonical NRPS Is Involved in the Synthesis of Fungisporin and Related Hydrophobic Cyclic Tetrapeptides in Penicillium chrysogenum
Source: PLoS One. 2014 Jun 2;9(6):e98212. doi: 10.1371/journal.pone.0098212 (PMC4041764; doi:10.1371/journal.pone.0098212)
Supplement: Table S2 — Primers designed for the expression analysis of the hcpA and actin gene. (DOCX) [file pone.0098212.s008.docx]

| Target | Primer sequence (5’- 3’) | | |
| --- | --- | --- | --- |
|  | Forward | Reverse | |
| *hcpA*  *actin* | GCTCGTGGCACCAGGTCCTGC CTGGCGGTATCCACGTCACC | | CGACTGGTGACCGTGTTTCGCC AGGCCAGAATGGATCCACCG |

**Table S2. Primers designed for the expression analysis of the *hcpA* and actin gene.**
