# Supplementary material for: A Non-Canonical NRPS Is Involved in the Synthesis of Fungisporin and Related Hydrophobic Cyclic Tetrapeptides in Penicillium chrysogenum
Source: PLoS One. 2014 Jun 2;9(6):e98212. doi: 10.1371/journal.pone.0098212 (PMC4041764; doi:10.1371/journal.pone.0098212)
Supplement: Table S3 — Retention time, formula and acquired m/z of cyclic and linear tetrapeptides obtained from metabolic profiling. Isomers have the same retention time as they could not be chromatographically separated during profiling. (DOCX) [file pone.0098212.s009.docx]

| peptide | peptide  sequence | RT | Formula | acquired mass [M+H]^+^ | accuracy |
| --- | --- | --- | --- | --- | --- |
|  |  | min |  | m/z | ppm |
| 1 | *cyclo-*(FFVV) | 27.33 | C28H36N4O4 | 493.2806 | -0.673 |
| 2 | *cyclo-*(YFVV) | 26.63 | C28H36N4O5 | 509.2757 | -0.288 |
| 3 | *cyclo-*(YWVV) | 26.59 | C30H37N5O5 | 548.2868 | 0.099 |
| 4 | *cyclo-*(FWVV) | 27.21 | C30H37N5O4 | 532.2916 | -0.434 |
| 5 | *cyclo-*(FFVI) | 27.56 | C29H38N4O4 | 507.2962 | -0.753 |
| 6 | *cyclo-*(FFIV) | 27.56 | C29H38N4O4 | 507.2962 | -0.753 |
| 7 | *cyclo-*(YWVI) | 26.86 | C31H39N5O5 | 562.3017 | -1.237 |
| 8 | *cyclo-*(YWIV) | 26.86 | C31H39N5O5 | 562.3017 | -1.237 |
| 9 | *cyclo-*(YFVI) | 26.90 | C29H38N4O5 | 523.2914 | -0.185 |
| 10 | *cyclo-*(YFIV) | 26.90 | C29H38N4O5 | 523.2914 | -0.185 |
| 11 | FVVF | 23.34 | C28H38N4O5 | 511.2912 | -0.580 |
| 12 | VFFV | 23.34 | C28H38N4O5 | 511.2912 | -0.580 |
| 13 | FFVV | 23.34 | C28H38N4O5 | 511.2912 | -0.580 |
| 14 | YFVV | 20.06 | C28H38N4O6 | 527.2864 | -0.022 |
| 15 | VYFV | 20.06 | C28H38N4O6 | 527.2864 | -0.022 |
| 16 | FVVY | 20.06 | C28H38N4O6 | 527.2864 | -0.022 |
| 17 | YWVV | 20.40 | C30H39N5O6 | 566.2972 | -0.195 |
| 18 | VYWV | 20.40 | C30H39N5O6 | 566.2972 | -0.195 |
| 19 | WVVY | 20.40 | C30H39N5O6 | 566.2972 | -0.195 |
| 20 | VFWV | 23.57 | C30H39N5O5 | 550.3023 | -0.174 |
| 21 | FWVV | 23.57 | C30H39N5O5 | 550.3023 | -0.174 |
| 22 | WVVF | 23.57 | C30H39N5O5 | 550.3023 | -0.174 |
| 23 | FVIF | 24.66 | C29H40N4O5 | 525.3077 | 1.053 |
| 24 | FIVF | 24.66 | C29H40N4O5 | 525.3077 | 1.053 |
| 25 | FVIY | 21.43 | C29H40O6N4 | 541.3023 | 0.441 |
| 26 | IYFV | 21.43 | C29H40O6N4 | 541.3023 | 0.441 |
| 27 | FIVY | 21.43 | C29H40O6N4 | 541.3023 | 0.441 |
| 28 | VYFI | 21.43 | C29H40O6N4 | 541.3023 | 0.441 |

**Table S3. Retention time, formula and acquired m/z of cyclic and linear tetrapeptides obtained from metabolic profiling.**

Isomers have the same retention time as they could not be chromatographically separated during profiling.
