# Supplementary material for: A Non-Canonical NRPS Is Involved in the Synthesis of Fungisporin and Related Hydrophobic Cyclic Tetrapeptides in Penicillium chrysogenum
Source: PLoS One. 2014 Jun 2;9(6):e98212. doi: 10.1371/journal.pone.0098212 (PMC4041764; doi:10.1371/journal.pone.0098212)
Supplement: Table S4 — 1H and 13C-NMR chemical shifts of synthetically produced compound 2 with sequence cyclo -( d -Tyr- l -Phe- d -Val- l -Val) in DMSO at 340 K. As synthetically and naturally produced 2 show identical NMR spectra, only chemical shifts for the synthetically produced compound are shown. δDMSO (1H/13C) = (2.55/40.50), (δ in ppm). (DOCX) [file pone.0098212.s010.docx]

| amino acid | position | δ (^1^H) |  | Position | δ(^13^C) |  |
| --- | --- | --- | --- | --- | --- | --- |
| *d*-Tyr | α | 4.53 |  | α | 54.10 |  |
|  | β1 | 2.93 |  | β | 34.59 |  |
|  | β2 | 2.69 |  | γ | 128.43 |  |
|  | δ | 6.99 |  | δ | 130.32 |  |
|  | ε | 6.65 |  | ε | 115.63 |  |
|  | NH | 7.82 |  | ζ | 156.35 |  |
|  | OH | 8.99 |  | C=O | 173.35 |  |
| *l*-Phe | α | 4.62 |  | α | 53.97 |  |
|  | β1 | 3.05 |  | β | 35.47 |  |
|  | β2 | 2.80 |  | γ | 138.48 |  |
|  | δ | 7.20 |  | δ | 129.41 |  |
|  | ε | 7.21 |  | ε | 128.68 |  |
|  | ζ | 7.26 |  | ζ | 126.72 |  |
|  | NH | 7.80 |  | C=O | 173.56 |  |
| *d*-Val | α | 4.00 |  | α | 59.90 |  |
|  | β | 2.00 |  | β | 27.66 |  |
|  | γ1 | 0.87 |  | γ1 | 19.76 |  |
|  | γ2 | 0.82 |  | γ2 | 19.01 |  |
|  | NH | 7.58 |  | C=O | 173.81 |  |
| *l*-Val | α | 3.98 |  | α | 59.71 |  |
|  | β | 2.00 |  | β | 27.51 |  |
|  | γ1 | 0.90 |  | γ1 | 19.73 |  |
|  | γ2 | 0.80 |  | γ2 | 19.07 |  |
|  | NH | 7.62 |  | C=O | 173.32 |  |

**Table S4.^1^H and ^13^C-NMR chemical shifts of synthetically produced compound 2 with sequence *cyclo*-(*d*-Tyr-*l*-Phe-*d*-Val-*l*-Val) in DMSO at 340 K.**

As synthetically and naturally produced **2** show identical NMR spectra, only chemical shifts for the synthetically produced compound are shown. δ_DMSO_ (^1^H/^13^C) = (2.55/40.50), (δ in ppm).
