# Supplementary material for: A Non-Canonical NRPS Is Involved in the Synthesis of Fungisporin and Related Hydrophobic Cyclic Tetrapeptides in Penicillium chrysogenum
Source: PLoS One. 2014 Jun 2;9(6):e98212. doi: 10.1371/journal.pone.0098212 (PMC4041764; doi:10.1371/journal.pone.0098212)
Supplement: Table S5 — 1H and 13C chemical shifts of naturally produced compound 1 with sequence cyclo -( d -Phe- l -Phe- d -Val- l -Val) present in a mix of various cyclic tetrapeptides in DMSO acquired at 340 K. Signals overlapping with the highest abundant compound 2 are indicated (*). δDMSO (1H/13C) = (2.55/40.50), (δ in ppm). (DOCX) [file pone.0098212.s011.docx]

| amino acid | position | δ (^1^H) |  | Position | δ(^13^C) |  |
| --- | --- | --- | --- | --- | --- | --- |
| *d*-Phe | α | 4.62* |  | α | 53.69 |  |
|  | β1 | 3.05* |  | β | 35.29 |  |
|  | β2 | 2.80* |  | γ | 138.40 |  |
|  | δ | 7.20* |  | δ | 129.43 |  |
|  | ε | 7.21* |  | ε | 128.66 |  |
|  | ζ | 7.26* |  | ζ | 126.74 |  |
|  | NH | 7.88 |  | C=O | 173.16 |  |
| *l*-Phe | Α | 4.62* |  | α | 54.03 |  |
|  | β1 | 3.05* |  | β | 35.56 |  |
|  | β2 | 2.80* |  | γ | 138.44 |  |
|  | δ | 7.20* |  | δ | 129.42 |  |
|  | ε | 7.21* |  | ε | 128.68* |  |
|  | ζ | 7.26* |  | ζ | 126.72* |  |
|  | NH | 7.78 |  | C=O | 173.56* |  |
| *d*-Val | α | 4.00* |  | α | 59.88 |  |
|  | β | 2.00* |  | β | 27.61 |  |
|  | γ1 | 0.86 |  | γ1 | 19.77 |  |
|  | γ2 | 0.81 |  | γ2 | 19.01* |  |
|  | NH | 7.58 |  | C=O | 173.79 |  |
| *l*-Val | α | 3.98* |  | α | 59.73 |  |
|  | β | 2.00* |  | β | 27.54 |  |
|  | γ1 | 0.89 |  | γ1 | 19.67 |  |
|  | γ2 | 0.78 |  | γ2 | 19.06 |  |
|  | NH | 7.58 |  | C=O | 173.36 |  |

**Table S5. ^1^H and ^13^C chemical shifts of naturally produced compound 1 with sequence *cyclo*-(*d*-Phe-*l*-Phe-*d*-Val-*l*-Val) present in a mix of various cyclic tetrapeptides in DMSO acquired at 340 K.**

Signals overlapping with the highest abundant compound **2** are indicated (*). δ_DMSO_ (^1^H/^13^C) = (2.55/40.50), (δ in ppm).
