# Supplementary material for: A Non-Canonical NRPS Is Involved in the Synthesis of Fungisporin and Related Hydrophobic Cyclic Tetrapeptides in Penicillium chrysogenum
Source: PLoS One. 2014 Jun 2;9(6):e98212. doi: 10.1371/journal.pone.0098212 (PMC4041764; doi:10.1371/journal.pone.0098212)
Supplement: Table S6 — 1H and 13C chemical shifts of isoleucine in compound 5 with sequence cyclo- (Phe-Phe-Val-Ile) and compound 9 with sequence cyclo- (Phe-Phe-Val-Ile) present in a extracted mix of cyclic tetrapeptides. NMR signals corresponding to C = O and NH as well as remaining amino acid signals are not observed (n.o.) due to overlap with the main constituents. δDMSO (1H/13C) = (2.55/40.50). (DOCX) [file pone.0098212.s012.docx]

| amino acid | position | δ (^1^H) |  | Position | δ(^13^C) |  |
| --- | --- | --- | --- | --- | --- | --- |
| Ile in **5** | α | 4.1 |  | α | 57.8 |  |
|  | β | 1.85 |  | β | 34.0 |  |
|  | γ1 | 0.76 |  | γ1 | 15.0 |  |
|  | γ2 | 1.3 |  | γ2 | 26.5 |  |
|  | γ2´ | 1.1 |  |  |  |  |
|  | δ | 0.9 |  | δ | 11.0 |  |
|  | NH | n.o. |  | C=O | n.o. |  |
| Ile in **9** | α | 4.1 |  | α | 57.5 |  |
|  | β | 1.85 |  | β | 33.5 |  |
|  | γ1 | 0.74 |  | γ1 | 16.0 |  |
|  | γ2 | 1.5 |  | γ2 | 24.5 |  |
|  | γ2´ | 1.15 |  |  |  |  |
|  | δ | 0.85 |  | δ | 10.5 |  |
|  | NH | n.o. |  | C=O | n.o. |  |

**Table S6. ^1^H and ^13^C chemical shifts of isoleucine in compound 5 with sequence *cyclo-*(Phe-Phe-Val-Ile) and compound 9 with sequence *cyclo-*(Phe-Phe-Val-Ile) present in a extracted mix of cyclic tetrapeptides.**

NMR signals corresponding to C=O and NH as well as remaining amino acid signals are not observed (n.o.) due to overlap with the main constituents. δ_DMSO_ (^1^H/^13^C) = (2.55/40.50).
