# Supplementary material for: A Non-Canonical NRPS Is Involved in the Synthesis of Fungisporin and Related Hydrophobic Cyclic Tetrapeptides in Penicillium chrysogenum
Source: PLoS One. 2014 Jun 2;9(6):e98212. doi: 10.1371/journal.pone.0098212 (PMC4041764; doi:10.1371/journal.pone.0098212)
Supplement: Table S7 — Sequencing data leading to the identification of linear tetrapeptides after AQC derivatization. Multiple-stage fragmentation was used for determination of b-ions for de-novo peptide sequencing. (DOCX) [file pone.0098212.s013.docx]

| linear peptide | underivatized  sequence | RT | formula derivatized peptide | [M+H]^+^ | b_3_ ion in MS^2^ | b_2_ ion in MS^3^ derived from b_3_ ion | b_1_ ion in MS^3^ derived from b_3_ ion |
| --- | --- | --- | --- | --- | --- | --- | --- |
|  |  | min |  | m/z | m/z (loss) | m/z (loss) | m/z (loss) |
| 11 | FVVF | 7.50 | C38H44N6O6 | 681 | 516 (-F) | 417 (-V) | 318 (-VV) |
| 12 | VFFV | 7.71 | C38H44N6O6 | 681 | 564 (-V) | 417 (-F) | 270 (-FF) |
| 13 | FFVV | 7.98 | C38H44N6O6 | 681 | 564 (-V) | 465 (-V) | 318 (-FV) |
| 14 | YFVV | 4.46 | C38H44N6O7 | 697 | 580 (-V) | 481 (-V) | 334 (-FV) |
| 15 | VYFV | 3.28 | C38H44N6O7 | 697 | 580 (-V) | 433 (-F) | 270 (-YF) |
| 16 | FVVY | 3.85 | C38H44N6O7 | 697 | 516 (-Y) | 417 (-V) | 318 (-VV) |
| 17 | YWVV | 4.20 | C40H45N7O7 | 736 | 619 (-V) | 520 (-V) | 334 (-WV) |
| 18 | VYWV | 2.55 | C40H45N7O7 | 736 | 619 (-V) | 433 (-W) | 270 (-YW) |
| 19 | WVVY | 3.64 | C40H45N7O7 | 736 | 555 (-Y) | 456 (-V) | 357 (-VV) |
| 20 | VFWV | 7.01 | C40H45N7O6 | 720 | 603 (-V) | 417 (-W) | 270 (-FW) |
| 21 | FWVV | 7.78 | C40H45N7O6 | 720 | 603 (-V) | 504 (-V) | 318 (-WV) |
| 22 | WVVF | 7.41 | C40H45N7O6 | 720 | 555 (-F) | 546 (-V) | 357 (-VV) |
| 23 | FVIF | 8.24 | C39H46N6O6 | 695 | 530 (-F) | 417 (-I) | 318 (-VI) |
| 24 | FIVF | 8.38 | C39H46N6O6 | 695 | 530 (-F) | 431 (-V) | 318 (-IV) |
| 25 | FVIY | 5.58 | C39H46N6O7 | 711 | 530 (-Y) | 417 (-I) | 318 (-VI) |
| 26 | IYFV | 4.62 | C39H46N6O7 | 711 | 594 (-V) | 447 (-F) | 284 (-YF) |
| 27 | FIVY | 6.14 | C39H46N6O7 | 711 | 530 (-Y) | 431 (-V) | 318 (-IV) |
| 28 | VYFI | 5.52 | C39H46N6O7 | 711 | 580 (-I) | 433 (-F) | 270 (-YF) |

**Table S7. Sequencing data leading to the identification of linear tetrapeptides after AQC derivatization.**

Multiple-stage fragmentation was used for determination of b-ions for de-novo peptide sequencing.
