# Supplementary material for: A Non-Canonical NRPS Is Involved in the Synthesis of Fungisporin and Related Hydrophobic Cyclic Tetrapeptides in Penicillium chrysogenum
Source: PLoS One. 2014 Jun 2;9(6):e98212. doi: 10.1371/journal.pone.0098212 (PMC4041764; doi:10.1371/journal.pone.0098212)
Supplement: Table S8 — Identified and proposed cyclic and linear tetrapeptides in respect to the adenylation domain specificity in the NRPS HcpA. (DOCX) [file pone.0098212.s014.docx]

| cyclic peptide | specificity of adenylation domain | | | | corresponding linear  peptide |
| --- | --- | --- | --- | --- | --- |
|  | A_1_ | A_2_ | A_3_ | A_4_ |  |
| 1 | Phe | Phe | Val | Val | 11-13 |
| 2 | Tyr | Phe | Val | Val | 14-16 |
| 3 | Tyr | Trp | Val | Val | 17-19 |
| 4 | Phe | Trp | Val | Val | 20-22 |
| 5 | Phe | Phe | Val | Ile | 23 |
| 6 | Phe | Phe | Ile | Val | 24 |
| 7 | Tyr | Trp | Val | Ile | *not observed* |
| 8 | Tyr | Trp | Ile | Val | *not observed* |
| 9 | Tyr | Phe | Val | Ile | 25-26 |
| 10 | Tyr | Phe | Ile | Val | 27-28 |
| *proposed* | Phe | Trp | Val | Ile | *not observed* |
| *proposed* | Phe | Trp | Ile | Val | *not observed* |
| *proposed* | Phe | Phe | Ile | Ile | *not observed* |
| *proposed* | Phe | Trp | Ile | Ile | *not observed* |
| *proposed* | Tyr | Phe | Ile | Ile | *not observed* |
| *proposed* | Tyr | Trp | Ile | Ile | *not observed* |
| specificity | Phe/Tyr | Phe/Trp | Val/Ile | Val/Ile |  |

**Table S8. Identified and proposed cyclic and linear tetrapeptides in respect to the adenylation domain specificity in the NRPS HcpA.**
